# Defining Metabolic Rewiring in Lung Squamous Cell Carcinoma

**DOI:** 10.3390/metabo9030047

**Published:** 2019-03-07

**Authors:** Rachel Paes de Araújo, Natália Bertoni, Ana L. Seneda, Tainara F. Felix, Márcio Carvalho, Keir E. Lewis, Érica N. Hasimoto, Manfred Beckmann, Sandra A. Drigo, Patricia P. Reis, Luis A. J. Mur

**Affiliations:** 1 Aberystwyth University, Institute of Biological, Environmental and Rural Sciences (IBERS), Ceredigion SY23 3DA, UK; rap23@aber.ac.uk (R.P.d.A.); meb@aber.ac.uk (M.B.); 2 São Paulo State University (UNESP), Faculty of Medicine, Dept. of Surgery and Orthopedics, Botucatu 18618687, Brazil; bertoni.na@gmail.com (N.B.); anaseneda@gmail.com (A.L.S.); felix.tainara@gmail.com (T.F.F.); ericanh80@hotmail.com (É.N.H.); sandradrigo@gmail.com (S.A.D.); 3 São Paulo State University (UNESP), Experimental Research Unity (UNIPEX), Botucatu 18618687, Brazil; 4 São Paulo State University (UNESP), School of Veterinary Medicine and Animal Science, Dept. of Veterinary Clinic, Botucatu 18618687, Brazil; marcio.carvalho@unesp.br; 5 Clinical Research Centre, Prince Philip Hospital, Hywel Dda University Health Board, Wales SA14 8QF, UK; k.e.lewis@swansea.ac.uk; 6 School of Medicine, Swansea University, Singleton Park, Swansea, Wales SA2 8PP, UK

**Keywords:** lung squamous cell carcinoma, flow infusion electrospray ionization high resolution mass spectrometry, untargeted metabolites, pathways

## Abstract

Metabolomics based on untargeted flow infusion electrospray ionization high-resolution mass spectrometry (FIE-HRMS) can provide a snap-shot of metabolism in living cells. Lung Squamous Cell Carcinoma (SCC) is one of the predominant subtypes of Non-Small Cell Lung Cancers (NSCLCs), which usually shows a poor prognosis. We analysed lung SCC samples and matched histologically normal lung tissues from eight patients. Metabolites were profiled by FIE-HRMS and assessed using *t*-test and principal component analysis (PCA). Differentially accumulating metabolites were mapped to pathways using the *mummichog* algorithm in R, and biologically meaningful patterns were indicated by Metabolite Set Enrichment Analysis (MSEA). We identified metabolic rewiring networks, including the suppression of the oxidative pentose pathway and found that the normal tricarboxylic acid (TCA) cycle were decoupled from increases in glycolysis and glutamine reductive carboxylation. Well-established associated effects on nucleotide, amino acid and thiol metabolism were also seen. Novel aspects in SCC tissue were increased in Vitamin B complex cofactors, serotonin and a reduction of γ-aminobutyric acid (GABA). Our results show the value of FIE-HRMS as a high throughput screening method that could be exploited in clinical contexts.

## 1. Introduction

The Global Cancer Statistics 2018 (based on GLOBOCAN 2018 estimates) indicate that lung cancer remains the leading cause of cancer incidence and mortality for 2018, with 2.1 million new cases and 1.8 million deaths [1]. Lung cancer can be classified into two main histologic types: non-small cell lung cancer (NSCLC, approximately 85% of the cases) or small cell lung cancer (SCLC, ~15%) [2]. NSCLC is further sub-classified into three main histological subtypes: adenocarcinoma (AD, ~40%), squamous cell carcinoma (SCC, ~30%), large cell carcinoma (~15%) and a few others [3]. Lung SCC is aggressive and commonly difficult to treat, as patients tend to be older and have a higher incidence of comorbidities compared to other NSCLC subtypes. Additionally, diagnosis tends to occur at an advanced disease stage [4]. New strategies are needed for patients with lung SCC, especially to understand the link between tumour behaviour and metabolism.

Metabolomics, the measurement of metabolites and metabolism as products of cellular processes [5] can be used in high through put mode to provide a “snap-shot” of metabolism, as represented by a biological sample [6,7]. Robust analyses pipelines have been developed for metabolomics, so that they are emerging as an informative technique to help precision medicine to further improve patient diagnosis and treatment [8,9]. Metabolism in lung cancer tumours is characterised by the Warburg effect, where even in the presence of normal levels of oxygen, glycolysis and the production of lactate is a predominant feature, even though this yields few molecules of ATP [10]. This leads to increases in glucose and TCA cycle intermediates [11,12]. Indeed, glucose activated proteins drive cancer proliferation [13]. Lung cancer carcinogenesis is also linked to perturbed lipid processing via β-oxidation so that reduced cytosolic levels of the long chain fatty acid catabolite acetylcarnithine is a marker for NSCLC [14]. The TCA cycle can be further fed by anaplerotic reactions from the metabolism of glutamine to form α-ketoglutarate, and amino acid processing is a feature of lung cancer. Increased glycolysis via glucose-6-phosphate also feeds into nucleotide and therefore nucleic acid biosynthesis, which can maintain tumour cell division [15]. Cellular membrane biosynthesis is fed by increased fatty acid production, leading to phospholipid production [16]. Thus, extensive biochemical characterisation has successfully yielded biomarkers for the detection and staging of lung cancers in tissue, serum, plasma and urine [17]. Our own analyses have detected potential biomarkers in sputum, and have suggested key differences in such as polyamine and lipid ganglioside metabolism [18].

Herein, we have analysed histologically paired normal and tissue samples from patients with lung SCC using flow infusion electrospray ionization high resolution mass spectrometry (FIE-HRMS) as an approach that allows rapid sample screening encompassing a wide range of metabolites. Our analyses indicated metabolic rewiring in lung SCC tumours that were broadly similar to other metabolomic studies of NSCLC. Some previously unreported features included changes in metabolism towards creatine, serotonin and a range of enzyme co-factors, most particularly, of the vitamin B complex. Our approach demonstrates the potential of FIE-HRMS in improving diagnosis and classification of tissues, and could be useful in the development of future clinical applications to improve targeted treatment strategies in lung SCC.

## 2. Materials and Methods

### 2.1. Patient Samples

This study was performed with the approval of the Research Ethics Board of the Faculty of Medicine, São Paulo State University (UNESP), Botucatu, São Paulo, Brazil. All eligible patients were untreated before surgery and provided informed consent before sample collection. A total of 8 paired samples (tumour tissue and matched histologically normal tissue) were collected from patients with lung SCC. Patients were primarily treated by surgical removal of the tumour, at Clinical Hospital of Botucatu in São Paulo state, Brazil. Primary lung SCC and histologically normal samples were subjected to frozen section histology analysis to confirm the presence of tumour or normal cells in at least 90% of tissues. Samples were snap-frozen in liquid nitrogen until metabolite extraction. Clinical and histopathological data are provided in Table 1.

### 2.2. Metabolites Extraction

The solid tumour and adjacent normal tissues (~10 mg) were placed in 2 mL sterile microcentrifuge tubes, each containing a single stainless steel ball (acetone cleaned), and were immediately flash-frozen in liquid N_2_ and homogenized using a ball mill, and then placed on ice. Then, 1 mL of chloroform:methanol:dH_2_O (1:2.5:1) was added. The samples were shaken at 4 °C degrees for 15 min and returned to ice. Samples were centrifuged at 4 °C degrees at 5000× *g* for 3 min and dried in vacuum to form a pellet in a microcentrifuge tube. The samples were couriered to the UK and analysed within 7d. To analyse the samples, 250 µL of 70 % methanol was added to the pellets which were resuspended by vortexing for 5 s. For flow infusion electrospray ionization high resolution mass spectrometry (FIE-HRMS), 100 µL of each sample was transferred into a glass vial and sealed. All samples were run in duplicate with no significant differences in the results obtained.

### 2.3. Untargeted Metabolite Fingerprinting by Flow Infusion Electrospray Ionization High Resolution Mass Spectrometry (FIE-HRMS)

FIE-HRMS was performed using Q executive plus mass analyser instrument with UHPLC system (Thermo Fisher Scientific©, Bremen, Germany), where *m/z* were generated in positive and negative ionization mode in a single run as described by Baptista et al. [19].

### 2.4. Statistical Analysis

Statistical analyses were performed with MetaboAnalyst 4.0 using R and Bioconductor packages [20]. Data filtering removed variables that were unlikely to be used when modelling the data based on the interquantile range (IQR) [21]. The data were normalised to percentage total ion count and then log transformed and auto scaled [22]. The univariate analyses used *t*-test to identify significant *m/z*, *p*-value > 0.05. Principal component analysis (PCA) and partial least squares-discriminant analysis (PLSDA) were used to distinguish the difference between the two sample groups. Major sources of variation were displayed using a heatmap and unsupervised hierarchical clustering.

The matched compound and pathway identification used the *mummichog* algorithm within MetaboAnalyst 4.0 from high-resolution MS peaks, without prior peak annotation. Compounds were identified based on mass-to charge (*m*/*z*); the *p*-values and t-scores which were used to interrogate the BioCyc library. Metabolite identification considered all potential matches (isotopes/adducts). The pathway enrichment tests used Fisher’s exact tests (FET) or hypergeometric *p*-values [23]. The metabolite and the disease interaction networks were based on KEGG, molecular interaction networks [24]. Metabolite Set Enrichment Analysis (MSEA) was performed to identify the biologically meaningful patterns [25]. Significant annotated compound names, Over Representation Analysis (ORA) and a metabolite set library were done as described [26,27].

## 3. Results

**Figure 1:**
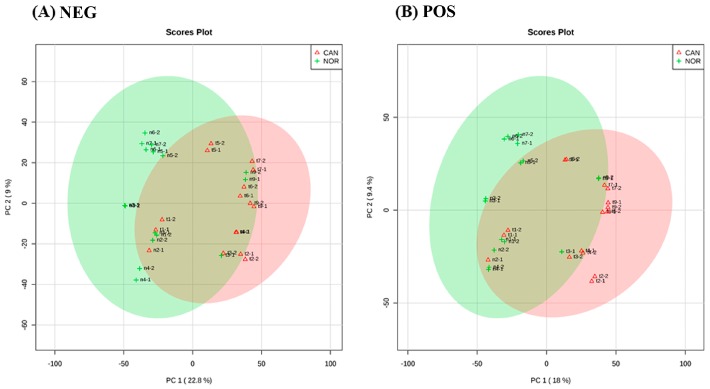
Principal component analysis (PCA) score plot between the selected PCs. The explained variances are shown in brackets, (**A**) is the negative ionization mode with a variation of 22.8% and (**B**) is the positive ionization mode with a variation of 18%, and the areas in red and green have 95% of confidence. The PCA shows distinction between both groups, normal tissue and cancer tissue.

In this study, 16 samples were assessed using FIE/MS-based metabolomics; 8 were lung cancer SCC tumour tissue, paired with 8 samples from the surrounding histologically normal tissue. The derived metabolomic data were assessed using PCA and for both negative (Figure 1A) and positive ionization mode (Figure 1B). The major sources of variation discriminate between the tumour and histologically normal tissue. Within the cancer group (green circles), no subdivisions into stages could be observed.

**Figure 2:**
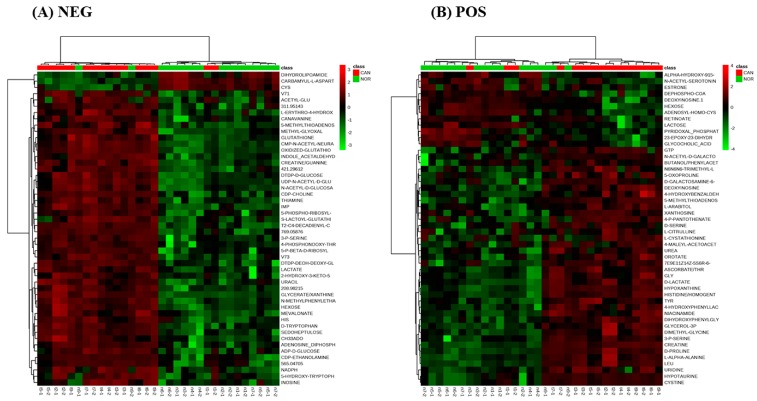
Hierarchical clustering analyses of negative ionization mode (**A**) and positive ionization mode (**B**) with top 50 differentially accumulated metabolites.

The major sources of variation were identified based on *t*-tests and corrected for false discovery rates (FDR) (Supplementary Tables S1 and S2). Unsupervised hierarchical cluster analysis again showed distinct metabolomics differences in normal vs tumour tissue (Figure 2A,B).

**Figure 3:**
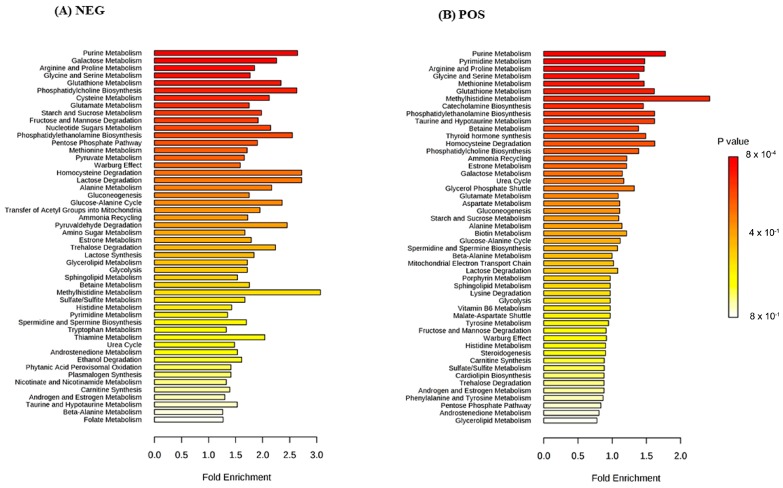
Significantly enriched pathways in SCC tumour tissue in (**A**) negative and (**B**) positive ionisation modes.

**Figure 4:**
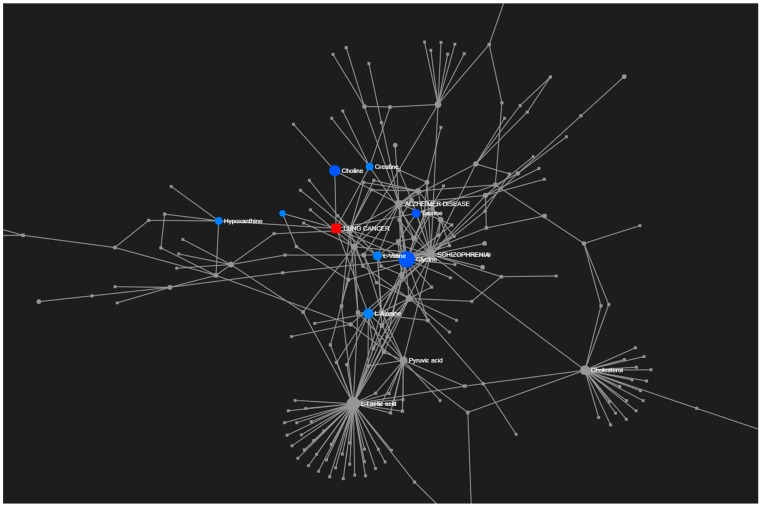
Joint-pathway analysis through an unbiased analyses linked our results to KEGG disease interaction focusing on lung cancer.

To provide an overview of the lung SCC metabolomes, the most significant metabolites were placed on to KEGG pathway maps. In both negative (Supplementary Figure S1) and positive ionization results (Supplementary Figure S2), the predominantly differentially responsive pathways were nucleotide metabolism, amino acid metabolism and glycolysis. The accumulation of most TCA metabolites was not detected in either ionization dataset. This was confirmed when data representing TCA metabolites were extracted from the matrix, and exhibited no significant differences between the paired tissue (Supplementary Figure S3). A Metabolite Set Enrichment Analysis (MSEA) based on Over Representation Analysis (ORA) was used to display significantly enhanced pathways (Figure 3). These indicated lung SCC-linked rewiring metabolism in nucleotide, sugar, amino acid and thiol pathways. Most of our characterised metabolite changes had already been linked to lung cancer, as also indicated by a joint-pathway analysis (Figure 4). Key nodes linked to hypoxanthine (nucleotide biosynthesis), choline (lipid processing), amino acids, taurine (thiol metabolism), and more indirectly to pyruvate, lactate and cholesterol.

**Figure 5:**
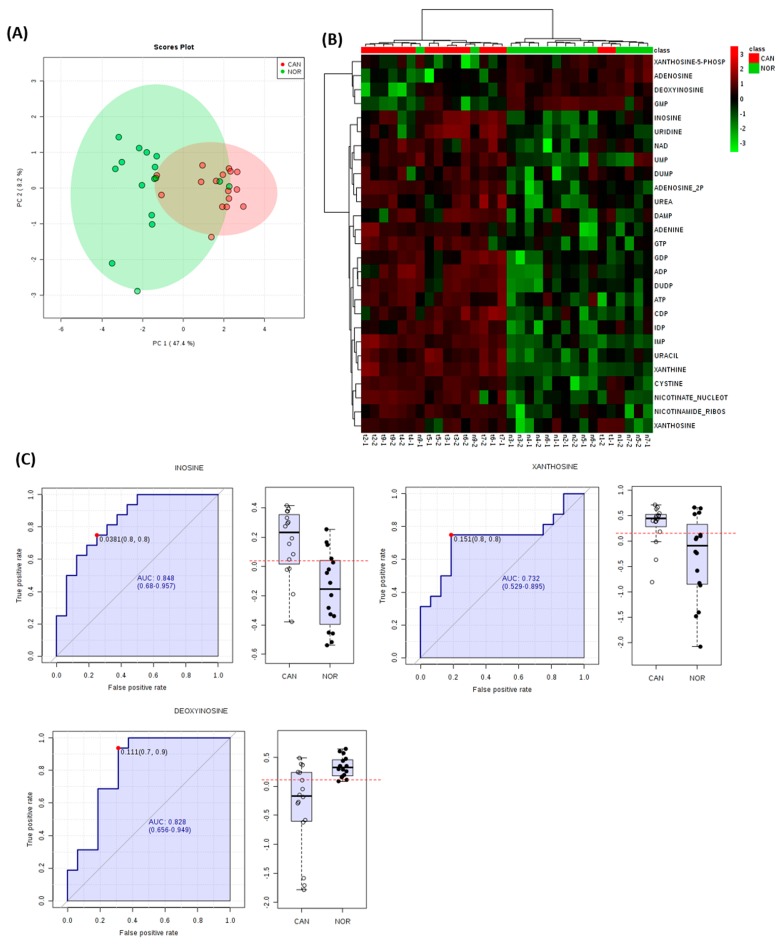
Altered nucleotide metabolism is a major feature of metabolism in lung SCC samples. Identified nucleotide metabolites for each sample were extracted from FIE-MS derived matrix and assessed by (**A**) principal component analyses and (**B**) hierarchical cluster analysis. (**C**) Area under the curve (AUC) and associated box and whisker plots of the main discriminatory nucleotide metabolites.

To gain further insights, the accumulation patterns of metabolites, which were significantly different in our analyses, were plotted within established pathways. Examination of nucleotide-associated metabolites indicated that differences in these were sufficient to discriminate between lung SCC and normal tissues in PCA and HCA (Figure 5A,B). Further, three individual metabolites exhibited AUC accuracies of >0.7 albeit with considerable variation (inosine, AUC= 0.848; CI 0.68-0.957, xanthosine, AUC = 0.732; CI 0.529-0.895, deoxyinosine, AUC= 0.828; CI 0.656–0.949) (Figure 5C).

**Figure 6:**
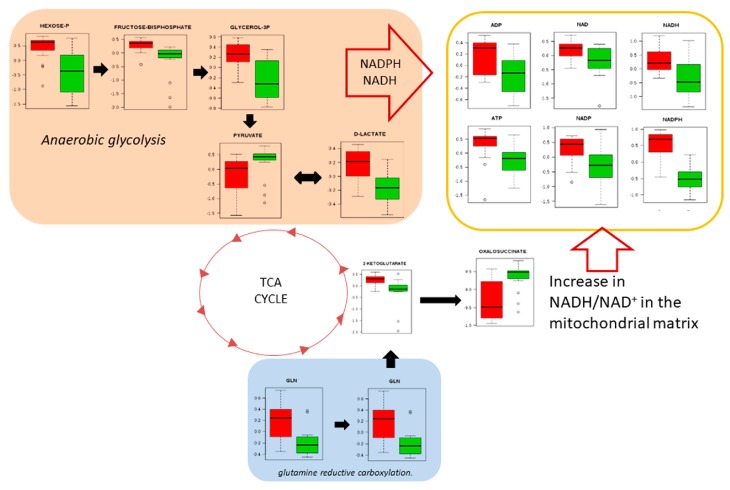
Anaerobic glycolysis is used to sustain glutamine reductive carboxylation. NAD(P)H electron transfer flux can sustain such as lipid synthesis. Red colours represent cancer tissue and green colours represent normal tissue.

Only slightly less prominent than nucleotides in our pathway analyses (Figure 3) was amino acid metabolism. This can be linked to the well-characterised generated of ATP and reducing equivalents that are required for growth and proliferation through the utilization of glucose to produce lactate, possibly through the glycolytic pathway and the reductive carboxylation of glutamate [28]. Alternatively/additionally, this could reflect a contribution by oxidative phosphorylation (Figure 6).

This is linked acetyl CoA driven lipogenesis, but was not prominent amongst the significantly metabolite changes (Supplementary Tables S1 and S2). However, it could reflect the activation of the mevalonate pathway leading to cholesterol biosynthesis, and increases in α-tocopherol accumulation (Vitamin E), which will preserve membrane integrity under oxidative stress conditions (Supplementary Figure S4). Other vitamin co-factors which would be required to maintain metabolism were also increased, except for B6 (pyridoxal phosphate). Ascorbate levels were increased in lung SCC, mostly likely in response to oxidative stress (Supplementary Figure S5).

Considering other changes in amino acid metabolism, important changes occurred in tryptophan catabolism (Supplementary Figure S6). In one branch of the pathway, increased tryptophan synthesis appeared to be driving the production of increased serotonin whilst melotonin levels were markedly reduced in lung SCC. In another branch, the synthesis of anti-inflammatory kyneurine was reduced, but there were increases in products, e.g., quiniolinate. Amino acid changes linked to the urea cycle were also affected with the accumulation of ornithine and arginine being severely reduced in tumours (Supplementary Figure S7). This inversely correlated with increased levels of urea and via ornithine to the polyamine spermidine. Notably, the levels of other polyamines (putrescine) and spermidine were severely reduced, as was GABA. The reduction in arginine was accompanied by an accumulation of creatine, but also by the absence of its less biologically-active derivative, creatinine. Another important aspect of glutamine/glutamate metabolism is, with cysteine, to feed into thiol metabolism (Supplementary Figure S8). In tumour samples, this apparently was linked to increases in glutathione, glutathione derivatives, (lactoyl- glutathione and hydroxylmethytl glutathione), and also taurine.

Pro-inflammatory events could be suggested by increases of the amino acid histidine through its metabolite histamine (Supplementary Figure S9). Such changes were paralleled by increases in free arachidonic acid, the precursor to eicosanoids such as prostaglandins. However, the levels of the core prostaglandin H2 were reduced, which could reflect further processing into other prostaglandins or sequestration of the increases of arachidonic acid into arachidonyl-CoA.

## 4. Discussion

Metabolomic approaches provide a comprehensive description of metabolism for a range of lung cancer types, offering the possibility of improved understanding of their aetiology, diagnosis and treatment. A change in the cellular metabolism is required for tumorigenesis, and studies have shown that cells need to generate an abundant amount of ATP for energy, along with de novo synthesis of nucleotides, lipids and proteins for fast proliferation [29]. Foundational metabolomic studies on lung cancer established alterations in the Krebs cycle, pyruvate carboxylation and lactate biochemistry, aligning with the perturbed bioenergy metabolism, which is a hallmark of cancer. Others soon considered differences between lung AD and SCC [30]. Such studies focused on a small number of patient samples, but a recent large metabolomic study based on 136 lung tissue samples has compared tumours and histologically normal tissue for each patient. Our study was smaller, with only samples from eight patients, paired with histologically normal samples and focused on SCC. However, whilst it confirmed many of the features of the Moreno et al. study by defining some new distinctive changes, it also demonstrated the value of FIE-MS in metabolomic studies of LC biopsies [31].

A range of metabolomic detection technologies have been employed in LC biopsy studies. These include nuclear magnetic resonance (NMR) [12], gas-chromatography MS (GC-MS) [31,32] and ultra-performance liquid chromatography (UPLC) linked to an Orbitrap Q Exactive Plus or linear trap quadrupole mass spectrometers. FIE-MS metabolomics offers the possibility of fast diagnoses, due to its high-throughput screening ability to capture a wide range of metabolites leading to reduced analysis times. Further, beyond a metabolite fingerprinting tool, when coupled with high resolution MS, FIE can provide some comprehensive descriptions of metabolites in a given sample to yield new insights. In this context, our FIE-MS-based analyses yielded large numbers of identified metabolites which could be identified based on high-resolution (3 ppm) data and ionisation patterns (Supplementary Tables S1 and S2), which significantly differed between SCC and histologically normal tissue. Further, analyses indicated changes in SCC which had been previously described in the literature (Supplementary Figure S3), but with novel features.

Warburg considered that in tumour cells, mitochondrial oxidative phosphorylation was permanently defective, making aerobic glycolysis the alternative and the main resource of energy for tumour cells [33]. For example, K-RAS activation in NSCLC cells was metabolically linked with the decoupling of glycolysis and TCA metabolism, with glutamine supplying increased carbon to drive the TCA cycle [34]. Although the genetic bases of our SCC samples were not defined, our results agree with such studies with the predominance of aerobic glycolysis, fed by glutamine/glutamate to feed the generation of ATP and reducing equivalents (Figure 6).

Mapping the significantly different metabolites on to KEGG metabolism indicated that alterations in nucleotide metabolism were predominant in lung SCC samples (Figure 3; Supplementary Figures S1 and S2). These observations agreed with the AD and SCC tumours, where nucleotide metabolism was suggested for therapeutic interventions and biomarkers (Moreau et al., 2018). The Moreau et al. study defined the top five nucleotides and metabolites which discriminated between AD and SCC tissue as indicated by AUC analysis. Most metabolites linked with SCC, but none of those with AD metabolites was also observed in our data with good AUC scores (Figure 6). Consistent with this, we observed increases in glycine, representing a major node in our network analysis (Figure 5). Glycine is a major source of methyl groups for the one-carbon pools, and is required for the biosynthesis of purines [35]. Glycine consumption and the expression of enzymes in the mitochondrial biosynthetic pathway correlate with the proliferation rate of cancer cells [36]. Glycine decarboxylase (GLDC) is crucial for tumour initiating cells in NSCLC, and was associated with pyrimidine metabolism to support cancer cell proliferation [37].

Another prominent change in lung SCC cells was the increases in the accumulation of most amino acids (Figure 3), represented by alanine and valine in our network analysis (Figure 4). One important regulatory node appears to be arginine/ornithine metabolism, which is important in supplying nitrogenous compounds to proliferating cancer cells [38]. Amongst our significant amino acid changes that could be linked to this urea cycle was a major reduction in both ornithine and arginine in SCC samples. This could reflect a rewiring of the metabolism toward urea and/or the production of NO via nitric oxide synthases with the co-production of citrulline (Supplementary Figure S7), and would support with a role of NO in tumour progression [39]. Arginine also appeared to be feeding into the production of creatinine, which apparently accumulated to the detriment of its catabolites, particularly creatinine (Supplementary Figure S7). Creatine forms part of an ATP buffering system comprised of the phosphocreatine (PCr)-creatine kinase (CK) shuttle, and has been linked to the Warburg effect and cancer progression [40].

Higher creatine levels have been seen in NSCLC when compared with normal tissue [41] and in SCLC primary tumours and cell cultures [42]. Lung AD and SCC tissues had lower CK activity, which accords with our elevated creatine levels [43], and was linked to a poor prognosis in LC patients [44]. Considering ornithine as a route to the production of polyamines, with the exception of spermidine, this was reduced. This differed from lung AD where spermine levels were elevated [45]. Also striking was a reduction in GABA, which is negative regulator of cell proliferation [46]. This agrees with the methylation and reduced expression of the GABA receptor GABBR2 in LC. Further, NSCLC patient with higher *GABBR2* expression had a better prognosis, possibly through links to EGFR signalling [47].

In the case of tryptophan metabolism, the effects appear to be seen in the accumulation of serotonin (5-hydroxytryptamine, 5-HT), to the detriment of melatonin. Serotonin has been suggested to be a growth factor in several cancers including SCLC, although not previously in lung SCC [48]. Serotonin has also been linked to angiogenesis and metastasis. In solid tumours, platelet aggregation can release serotonin, which may constitute one of the mechanisms of tumour progression and angiogenesis, and could result in higher serotonin levels in the blood [49].

Taurine also formed a node in our network model (Figure 4); this was indicative of wider effect on thiol metabolism (Supplementary Figure S8). Cysteine metabolism partly fed into increases in hypotaurine and taurine in SCC samples. This was unlike decreases observed in taurine in the lung adenocarcinoma cell line A549, which allowed us to conclude that taurine can inhibit cell proliferation [50], or that levels of taurine are reduced in patients with breast cancer [51]. Additionally, cysteine fed into glutathione production excess of the latter promotes tumour progression, where high levels are correlated with increased metastasis [52]. Glutathione also seemed to be feeding into the glyoxalase system in an attempt to remove methylglyoxal as a side product of anaerobic glycolysis. Increases in methylglyoxal lead to genomic damage [53]. The formation of S-lactoyl glutathione is catalysed by glyoxalase 1 (Glo1) and under-expression can promote tumour growth [54]. Perhaps counter-intuitively, over expression of Glo1 is a biomarker for tumour growth, but this is likely to reflect high-glycolytic activity, as we observed in SCC tissues [55].

Other well-established features were also observed in our lung SCC metabolomes. Choline is a marker for lipid processing and was key network node in our analyses (Figure 4). Abnormalities in choline metabolism are emerging as a metabolic hallmark of oncogenesis and tumour progression [50]. Increases in cholesterol are components of increased lipid biosynthesis to support the production of membranes [56]. Cholesterols are essential to the formation of lipid rafts and are platforms of oncogenic activation of PI3K-Akt-mTORC1 [57] or EGRF [58], with the efficacy of tyrosine kinase inhibitors being affected by EGFR location to lipid rafts. Key metabolites of the mevalonate pathway accumulated in SCC tumours, as well-established in cancer tissues, to feed into, amongst other things, cholesterol [59].

Other notable changes included increases in a range of vitamins and cofactors that are indicative of increased cellular metabolism, some of which have not been previously reported in lung SCC tissue. Thus, vitamin B1 (thiamine) plays an essential role in key enzyme of the pentose phosphate pathway and amino acid catabolism [60]. Increases in B1, linked to increased expression of the transporter (THTR2), have been reported in breast cancer, and chemotherapeutic drugs such as 5-fluorouracil (5-FU) result in a thiamine-deficient state [61,62]. The role of thiamine appears to be dose dependent, with low doses serving to promote, but with high doses suppressing proliferation [63]. Vitamin B2 (riboflavin) is a cofactor for methylenetetrahydrofolate reductase (MTHFR), which is important for nucleotide production. Treatment with high dose riboflavin could enhance lung carcinogenesis, but increases have not been linked to SCC tumours [64].

Pyridoxal 5’-phosphate (PLP), the active form of vitamin B6, works as cofactor in numerous enzymatic reactions; it behaves as antioxidant molecule [65]. Unlike other cofactors, deficiencies in PLP are seen in cancer tissues including lung cancer [66,67], and this may be linked to antioxidant and maintenance of genome integrity [65]. This may reflect the role of PLP as a cofactor for SHMT, which catalyzes the reversible conversion of serine and tetrahydrofolate to feed into nucleotide biosynthesis. Thus, reduced PLP links with corresponding increases in nucleotide and serine production.

Perturbation of the inflammatory response is a cancer hallmark, but our study yielded equivocal results. Histidine feeding through to histamine could be linked to pro-inflammatory events, but arachidonic acid levels and those of the central intermediate eiconsanoid biosynthesis were suppressed. The kynurenine pathway can be involved in immune suppression, and can contribute to immune privilege in cancer microenviroments [68]. However, this pathway was not significant in SCC tumours, with any changes possibly feeding into NAD^+^ biosynthesis (Supplementary Figure S6).

## 5. Conclusions

Our analyses show that untargeted FIE- MS represents an appropriate approach to develop an understanding of the metabolic process of growth and proliferation in SCC. This is demonstrated through our observation of well-characterised biochemical pathways in lung cancer pathways. Our findings give a general idea of the metabolites that are in the metabolic pathways of nucleotide metabolism, amino acid metabolism and energy metabolism, which are important pathways that provide the necessary supplies for tumour growth and proliferation. The ability of FIE-MS to indicate novel aspects was also highlighted in our work. We demonstrate some features in SCC samples which have yet to be described in this tissue type, including changes in vitamin cofactors, serotonin and a reduction in GABA (a putative tumour suppressor). Given the high throughput potential of FIE-MS, it is possible to apply such approaches in strategies focusing on personalised medicine.

## Figures and Tables

**Table 1:** Clinical and histopathological characteristics of samples (8 patients).

Variables	N
**Age at diagnosis (years)**	
Median (range)	55 (39–71)
**Gender**	
Male	5
Female	3
**Tobacco use**	
Yes	7
No	1
**Alcohol use**	
Yes	5
No	3
**T category**	
T1-T2	6
T3-T4	2
**Nodal status (pathological)**	
Negative (N0)	6
Positive (N1, N2)	2
**Disease stage**	
IA	1
IIA	1
IIIA	2
IB	3
IIB	1
**Tumour grade**	
Well differentiated	1
Moderately differentiated	5
Poorly differentiated	2
**Perineural invasion**	
Yes	1
No	7
**Angiolymphatic invasion**	
Yes	6
No	2
**Outcome**	
Alive with disease	6
Death cause by disease	2

## Supplementary Materials

The following are available online at https://www.mdpi.com/2218-1989/9/3/47/s1, Figure S1, Figure S2, Figure S3, Figure S4, Figure S5, Figure S6, Figure S7, Figure S8, Figure S9, Table S1, Table S2.
