# Supplementary material for: Defining Metabolic Rewiring in Lung Squamous Cell Carcinoma
Source: Metabolites. 2019 Mar 7;9(3):47. doi: 10.3390/metabo9030047 (PMC6468359; doi:10.3390/metabo9030047)
Supplement: Supplementary file 1 [file metabolites-09-00047-s001.pdf]

metabolites

Supplementary Materials

Defining Metabolic Rewiring in Lung Squamous Cell Carcinoma

Rachel Paes de Araújo [1], Natália Bertoni [2,3], Ana L. Seneda [2,3] Tainara F. Felix [2,3]; Márcio Carvalho [4]; Keir E. Lewis [5,6]; Érica N. Hasimoto [2]; Manfred Beckmann [1]; Sandra A. Drigo [2,3]; Patricia P. Reis [2,3,*] and Luis A. J. Mur [1,*]

[1] Aberystwyth University, Institute of Biological, Environmental and Rural Sciences (IBERS), Ceredigion SY23 3DA UK; rap23@aber.ac.uk (R.P.d.A.); meb@aber.ac.uk (M.B.)

[2] São Paulo State University (UNESP), Faculty of Medicine, Dept. of Surgery and Orthopedics, 18618687 Botucatu, Brazil. bertoni.na@gmail.com(N.B.); anaseneda@gmail.com(A.L.S.); felix.tainara@gmail.com (T.F.F.) ericanh80@hotmail.com (E.N.H.)sandradrigo@gmail.com (S.A.D.)

[3] São Paulo State University (UNESP), Experimental Research Unity (UNIPEX), 18618687 Botucatu, Brazil.

[4] São Paulo State University (UNESP), School of Veterinary Medicine and Animal Science, Dept. of Veterinary Clinic, 18618687 Botucatu, Brazil. marcio.carvalho@unesp.br (M.C.)

[5] Clinical Research Centre, Prince Philip Hospital, Hywel Dda University Health Board, Wales SA14 8QF, UK; k.e.lewis@swansea.ac.uk (K.E.L.)

[6] School of Medicine, Swansea University, Singleton Park, Swansea, Wales SA2 8PP, UK

* Correspondence: lum@aber.ac.uk (L.A.J.M.); patricia.reis@unesp.br (P.P.R.); Tel.: + 44 (0)1970 (L.A.J.M.); +55 (14) 3880-1451 (P.P.R.)

Received: 8 February 2019; Accepted: 2 March 2019; Published: 7 March 2019

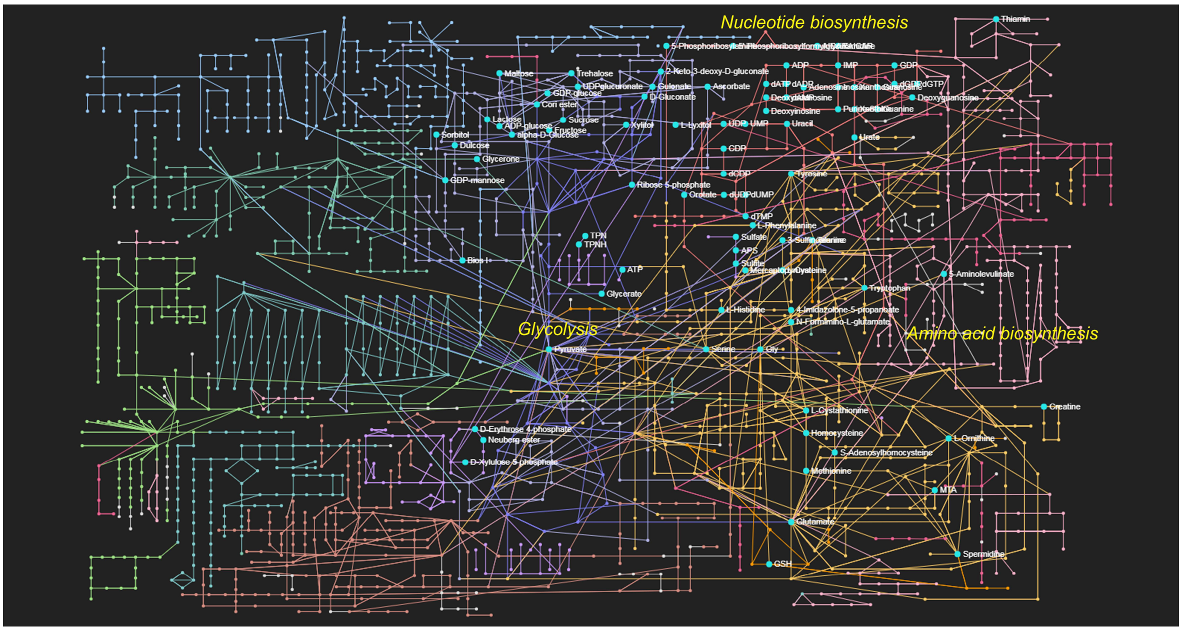

Supplementary Figure S1: Metabolites indicated by FIE-MS in negative ionisation mode that discriminate between lung squamous cell carcinoma and pair histologically normal tissue mapped on KEGG whole metabolism maps. The most significant compounds are indicated with light blue nodes.

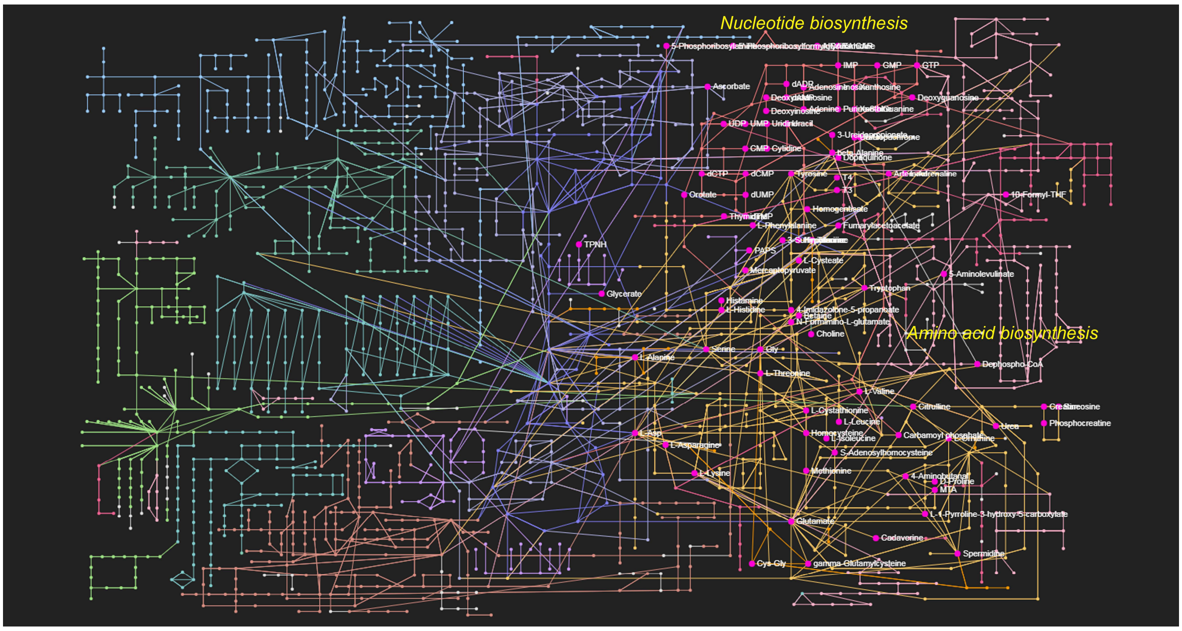

Supplementary Figure S2: Metabolites indicated by FIE-MS in positive ionisation mode that discriminate between lung squamous cell carcinoma and pair histologically normal tissue mapped on KEGG whole metabolism maps. The most significant compounds are indicated with pink nodes.

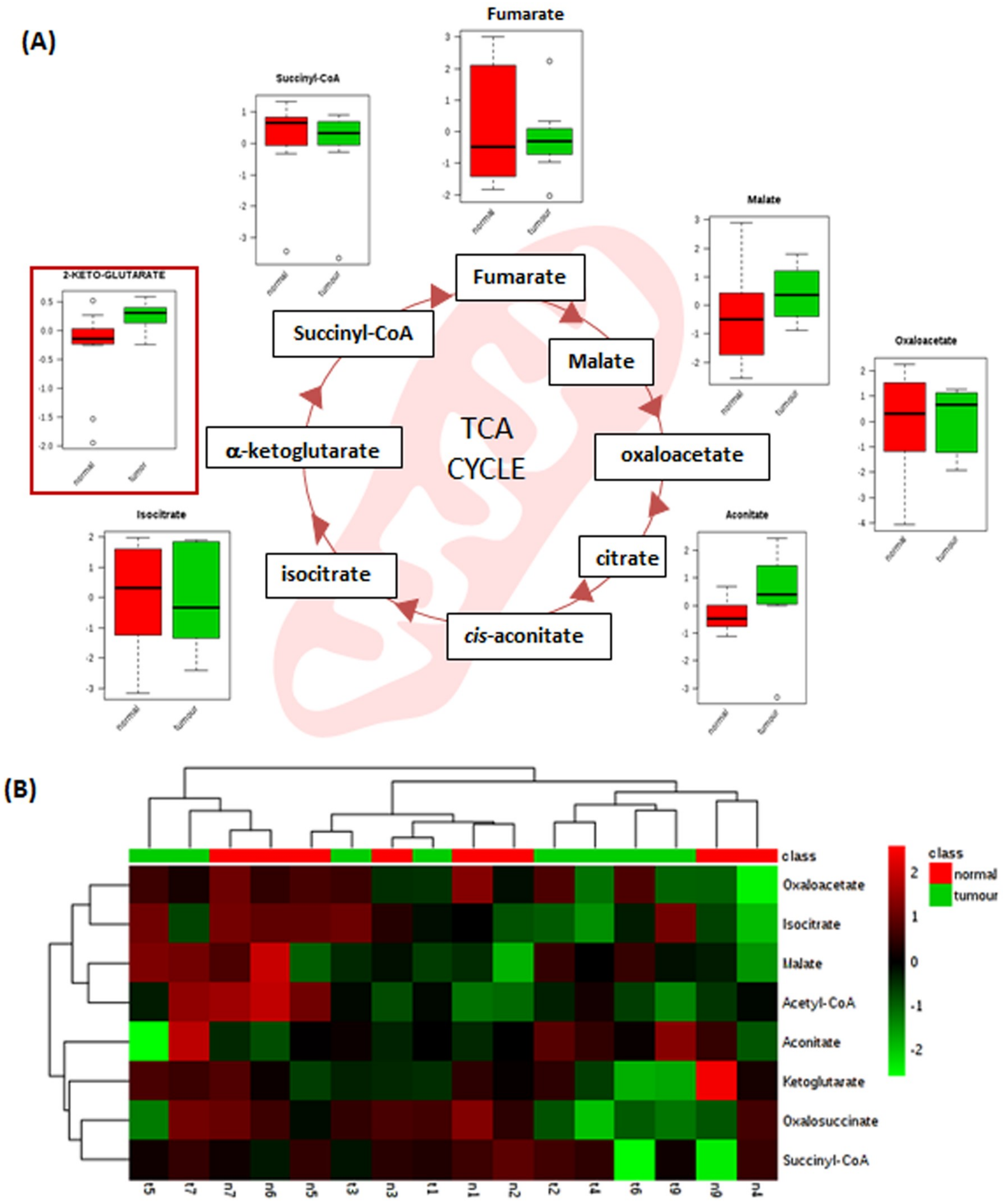

Supplementary Figure S3: TCA metabolite accumulation in lung squamous cell carcinoma (SCC) and paired samples **(A)** plotted as relative levels (only a-ketoglutarate; boxed in red, was significantly increased in SCC tissue) or **(B)** compared using hierarchical cluster analysis. No SSC associated clustering was observed.

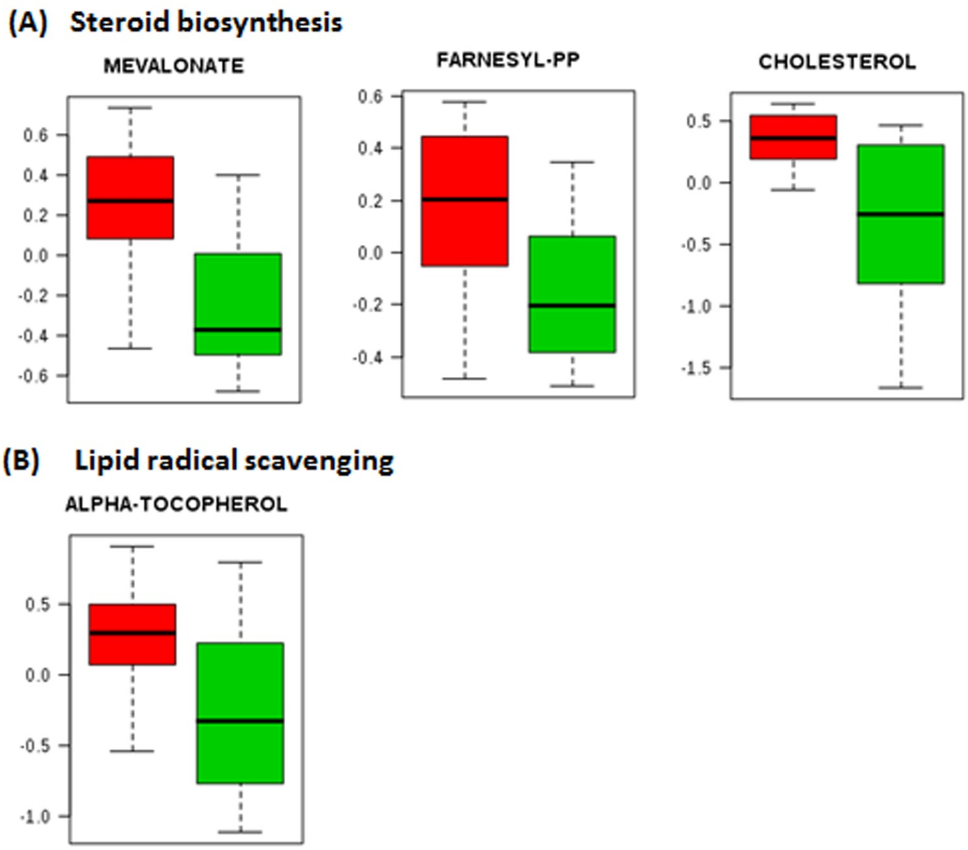

Supplementary Figure S4: Membrane associated metabolites significantly increased in lung SCC samples (red) versus paired histologically normal controls (green).

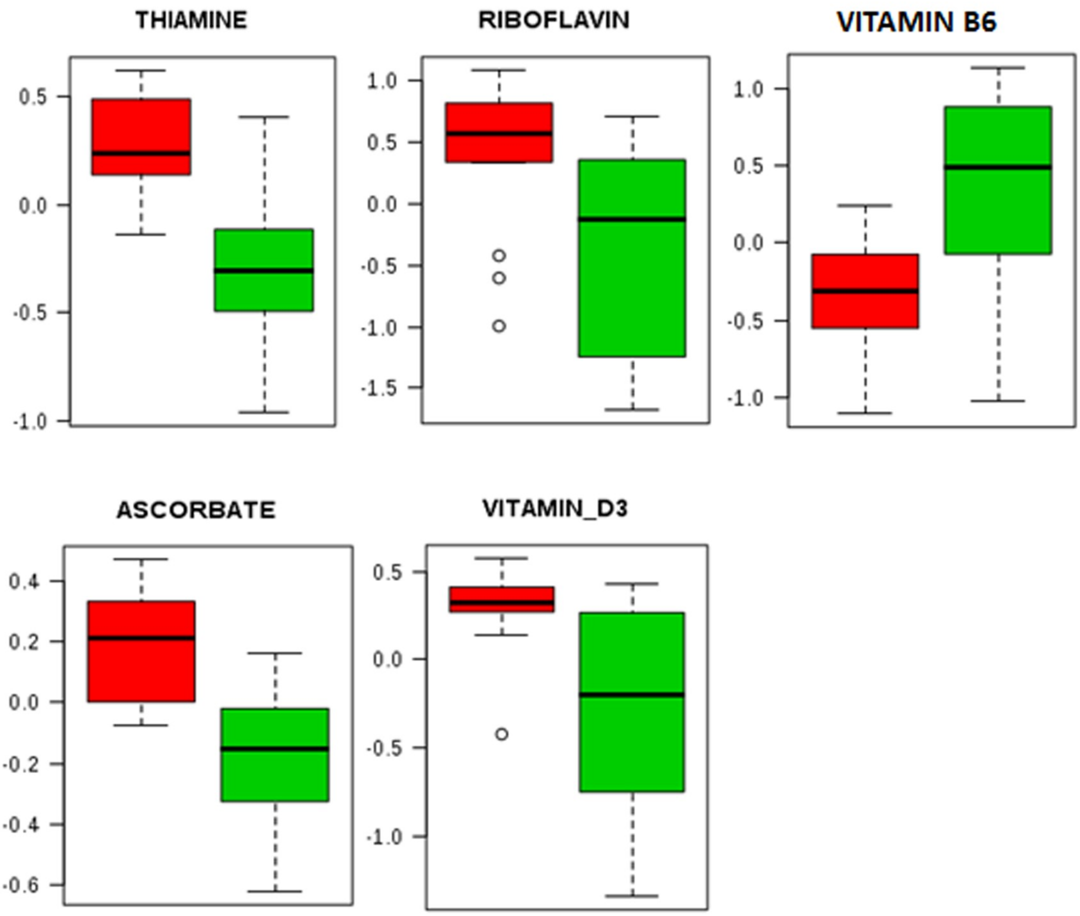

Supplementary Figure S5: Vitamin and Enzyme co-factor increases in lung SCC samples (red) versus paired histologically normal controls (green).

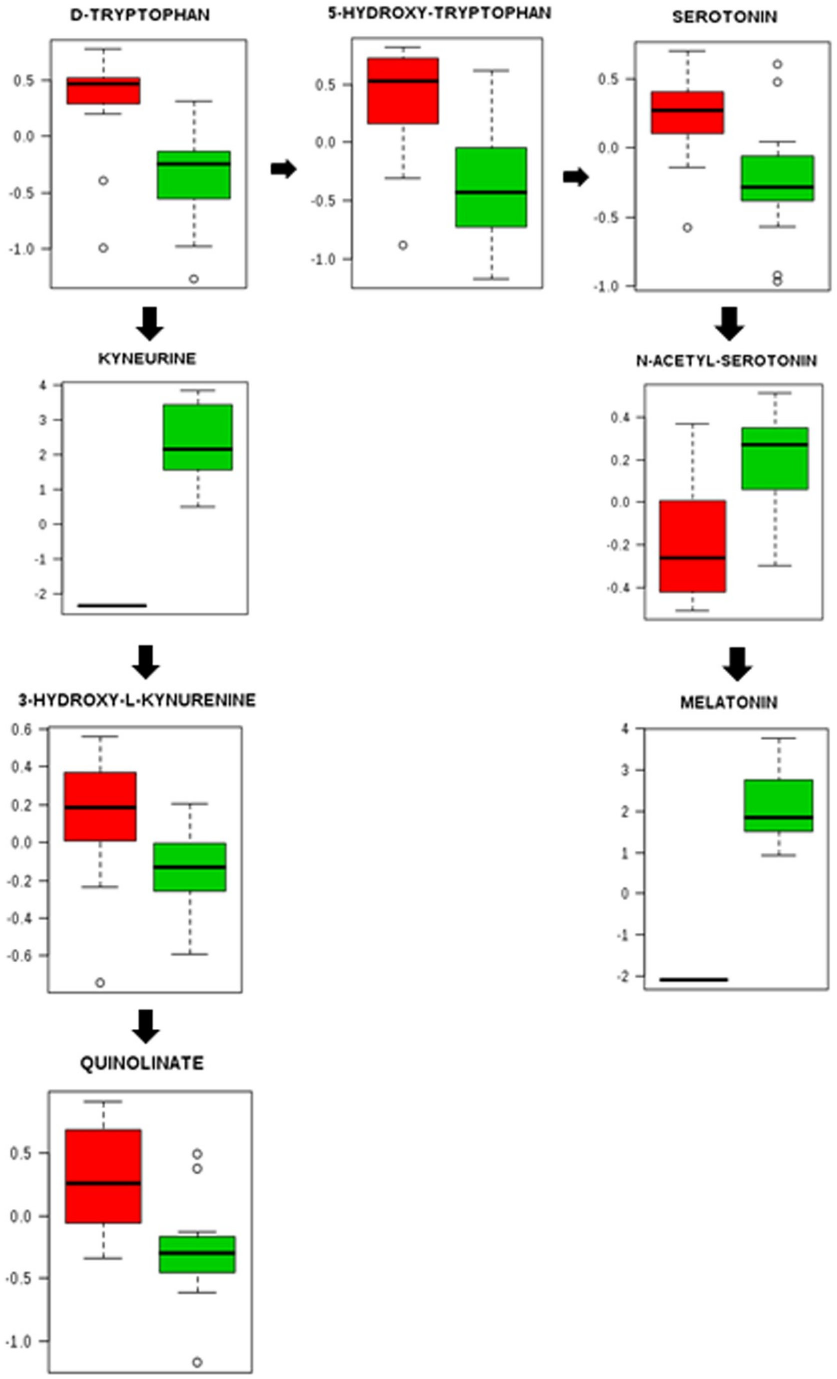

Supplementary Figure S6: Changes in tryptophan metabolism in lung SCC samples (red) versus paired histologically normal controls (green).

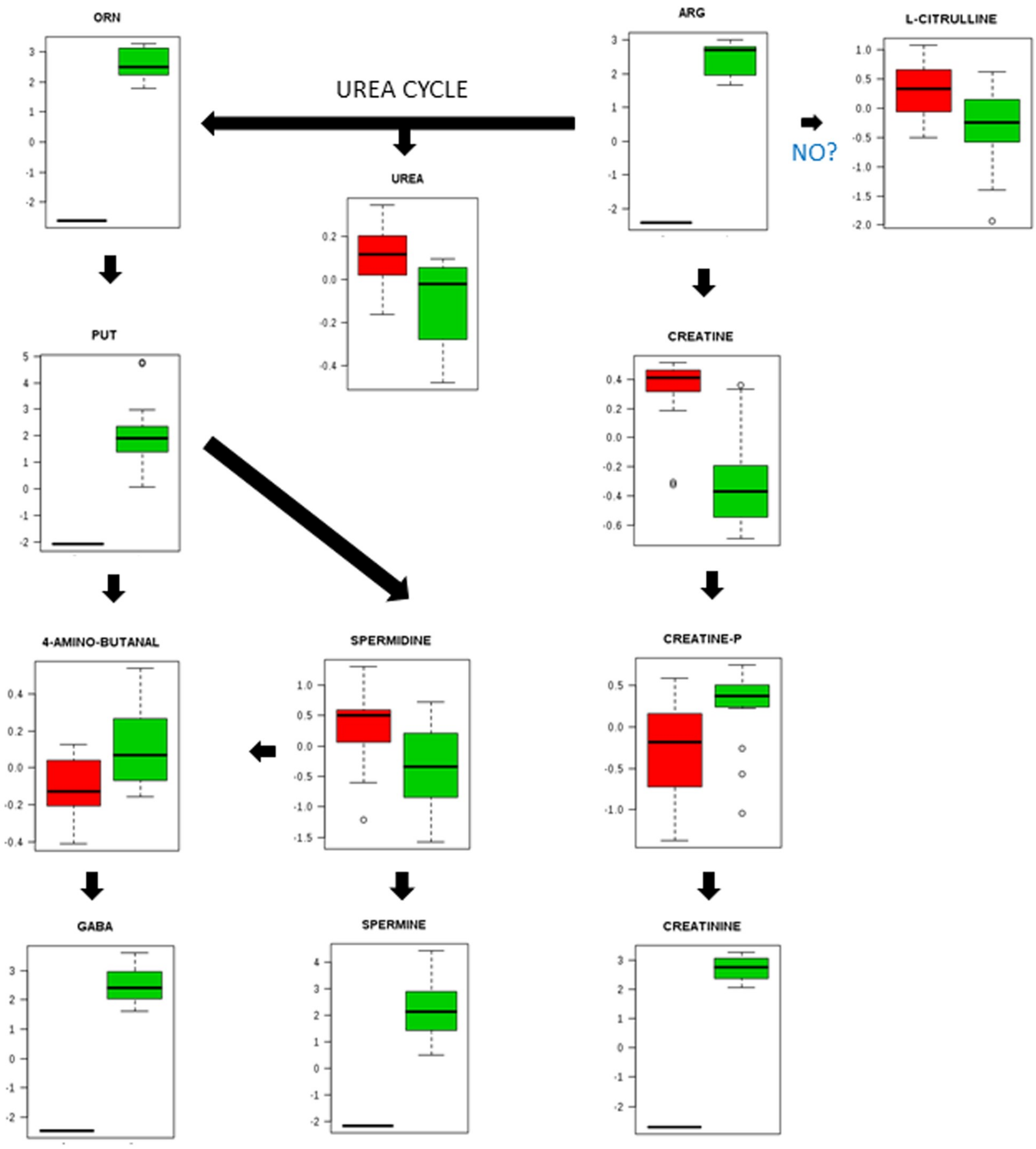

Supplementary Figure S7: Changes in urea cycle outputs in lung SCC samples (red) versus paired histologically normal controls (green). A possible link between arginine (ARG) and L-citrulline via the production of nitric oxide from nitric oxide synthase.

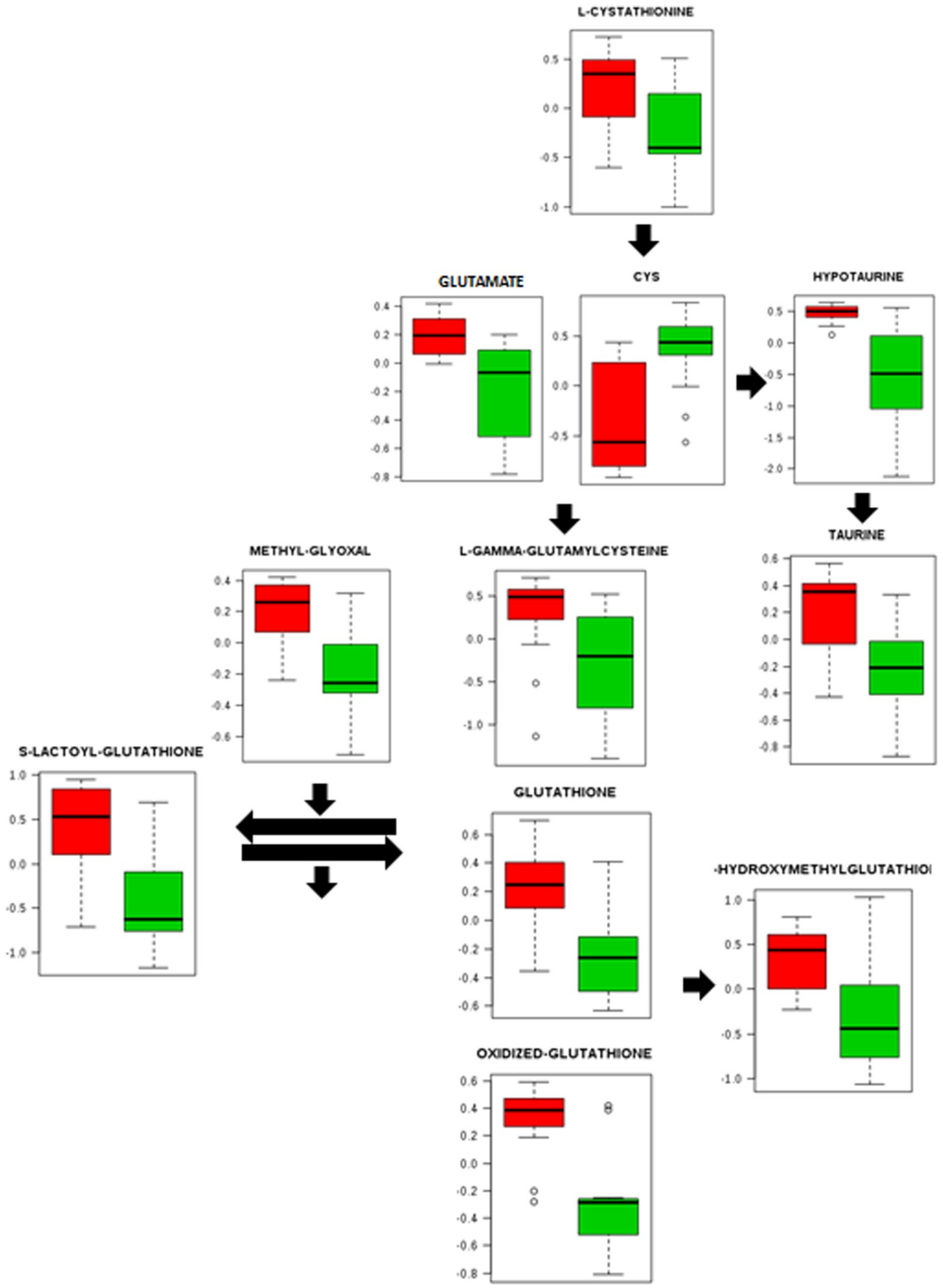

Supplementary Figure S8: Changes in thiol metabolism in lung SCC samples (red) versus paired histologically normal controls (green).

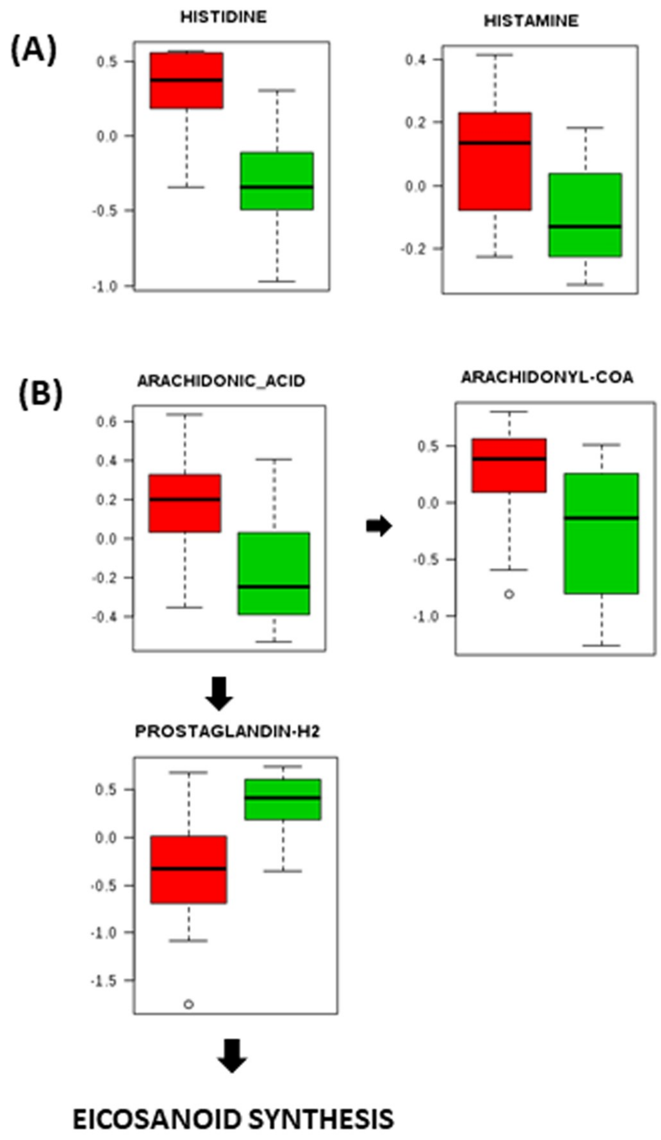

Supplementary Figure S9: Putative changes in metabolism in lung SCC samples (red) versus paired histologically normal controls (green) that could be linked to inflammatory events. **(A)** The synthesis of histamine from histidine is increased in SSC samples. **(B)** Increases in arachidonic acid do not correlate with increases in the eicosanoid intermediate prostaglandin H2 but with the formation of the archidonyl-CoA.

Supplementary Table S1: Positive ionization annotated compounds with their correspondent p.value, FDR and their expression.

Compound	p.value	FDR	CAN	NOR
DIHYDROXYPHENYLGLYCOLALDEHYDE	3.48E-07	2.05E-05	High	Low
HYPOXANTHINE	3.91E-07	2.05E-05	High	Low
CREATINE	5.04E-07	2.05E-05	High	Low
OROTATE	2.44E-06	6.05E-05	High	Low
HYPOTAURINE	2.48E-06	6.05E-05	High	Low
D-PROLINE	3.87E-06	7.34E-05	High	Low
HISTIDINE/HOMOGENTISATE	4.21E-06	7.34E-05	High	Low
LEUCINE	7.99E-06	0.000121	High	Low
CYSTINE	8.93E-06	0.000121	High	Low
L-ALPHA-ALANINE	1.08E-05	0.000131	High	Low
4-HYDROXYPHENYLLACTATE	1.35E-05	0.00015	High	Low
ASCORBATE/THR	1.89E-05	0.000192	High	Low
3-PHOSPHO-L-SERINE	4.20E-05	0.000395	High	Low
GLYCEROL-3P	5.10E-05	0.000444	High	Low
DIMETHYL-GLYCINE	5.55E-05	0.000451	High	Low
TRIMETHYLOLPROPANE	6.04E-05	0.000461	High	Low
URIDINE	7.30E-05	0.000524	High	Low
5-METHYLTHIOADENOSINE	8.26E-05	0.000557	High	Low
TYROSINE	8.87E-05	0.000557	High	Low
NIACINAMIDE	9.37E-05	0.000557	High	Low
23-EPOXY-23-DIHYDRO-2-METHYL-14-NAPHTHOQ	9.59E-05	0.000557	Low	High
GLYCINE	0.000105	0.000582	High	Low
L-GLUTAMATE	0.000187	0.000991	High	Low
4-P-PANTOTHENATE	0.000247	0.001254	High	Low
HEXOSE	0.000278	0.001325	Low	High
CALCIDIOL	0.000284	0.001325	High	Low
DIHYDRO-THYMINE	0.000293	0.001325	High	Low
D-LACTATE	0.000311	0.001357	High	Low
ADENOSYL-HOMO-CYS	0.000345	0.001451	Low	High
VALINE	0.000408	0.00166	High	Low
N-ACETYL-D-GALACTOSAMINE-6-PHOSPHATE	0.000449	0.00174	High	Low
5-FORMYL-THF	0.000462	0.00174	Low	High
ALPHA-HYDROXY-915-DIOXOPROSTANOATE	0.000471	0.00174	Low	High
QUINOLINATE	0.000501	0.001761	High	Low
PYRIDOXAL_PHOSPHATE	0.000505	0.001761	Low	High
N-ACETYL-SEROTONIN	0.000532	0.001802	Low	High
DEOXYINOSINE	0.00055	0.001812	High	Low
DEOXYINOSINE.1	0.000577	0.00184	Low	High

INOSINE	0.000588	0.00184	High	Low
ALBENDAZOLE	0.000839	0.002559	High	Low
TAURINE	0.000955	0.002825	High	Low
N1-METHYLADENINE	0.000987	0.002825	High	Low
METHYLARSONITE	0.000996	0.002825	Low	High
ALPHA-GLUCOSE-16-BISPHOSPHATE	0.001089	0.00302	High	Low
ESTRONE	0.001341	0.003635	Low	High
NICOTINATE_NUCLEOTIDE	0.001428	0.00369	High	Low
UREA	0.001445	0.00369	High	Low
D-SERINE	0.001452	0.00369	High	Low
5-AMINOIMIDAZOLE-4-CARBOXAMIDE RIBONUCLEOTIDE	0.001488	0.003705	High	Low
BUTANOL/PHENYLACETATE	0.001589	0.003876	High	Low
L-OCTANOYLCARNITINE/L-PIPECOLATE	0.001703	0.004075	High	Low
ALPHA-TOCOPHEROL	0.001745	0.004093	High	Low
LACTOSE	0.001789	0.004114	Low	High
BETA-HYDROXYANDROST-5-EN-17-ONE-3-SULFAT	0.001853	0.004114	High	Low
ADENINE	0.001866	0.004114	High	Low
7E9E11Z14Z-5S6R-6-CYSTEIN-S-YL	0.001888	0.004114	High	Low
ARACHIDONIC_ACID	0.001968	0.004149	High	Low
METHIONINE	0.001973	0.004149	High	Low
GLUTAMINE	0.002009	0.004154	High	Low
OXIDIZED-DITHIOTHREITOL	0.002238	0.004537	High	Low
N2-SUCCINYLGLUTAMATE	0.002269	0.004537	Low	High
4-HYDROXYBENZALDEHYDE	0.002348	0.004619	High	Low
TRYPTOPHAN	0.002385	0.004619	High	Low
NEUROSPORENE	0.002464	0.004662	Low	High
TAUROLITHOCHOLATE-SULFATE	0.002522	0.004662	High	Low
4-MALEYL-ACETOACETATE	0.002522	0.004662	High	Low
B-ALANINE	0.002659	0.004777	High	Low
OXIDIZED-GLUTATHIONE	0.002663	0.004777	Low	High
RETINOATE	0.002769	0.004791	Low	High
DEPHOSPHO-COA	0.002784	0.004791	Low	High
GUANOSINE TRIPHOSPHATE	0.002796	0.004791	High	Low
URACIL	0.002828	0.004791	High	Low
D-GALACTOSAMINE-6-PHOSPHATE	0.002931	0.004898	High	Low
4-AMINO-BUTYRALDEHYDE	0.00299	0.004929	Low	High
D-SEDOHEPTULOSE-7-P	0.003085	0.005019	High	Low
BETA-D-GALACTOSYL-ETCETERA-GLUCOSAMINE	0.003181	0.005106	High	Low
ACETYL-ETCETERA-L-ASPARAGINE	0.003251	0.005151	High	Low
L-GAMMA-GLUTAMYLCYSTEINE	0.003393	0.005307	High	Low
GLYCOCHOLIC_ACID	0.003482	0.005312	Low	High

SUCROSE	0.003523	0.005312	High	Low
5-OXOPROLINE	0.003527	0.005312	High	Low
GLYCERATE/XANTHINE	0.003579	0.005325	High	Low
BETA-D-XYLOSE	0.003764	0.005505	High	Low
L-CITRULLINE	0.003805	0.005505	High	Low
RIBOFLAVIN/SARCOSINE	0.003836	0.005505	High	Low
L-CYSTATHIONINE	0.004012	0.005652	High	Low
GUANOSINE MONOPHOSPHATE	0.004031	0.005652	Low	High
BUTANAL	0.00414	0.005739	Low	High
DIETHYLTHIOPHOSPHATE	0.00439	0.006017	High	Low
THREONINE	0.004439	0.006017	High	Low
PHENYLALANINE	0.004569	0.006125	High	Low
ACETONE	0.004711	0.006183	Low	High
PYRIDOXAL	0.004713	0.006183	High	Low
3-SULFINOALANINE	0.004891	0.006348	High	Low
BILIVERDINE	0.005282	0.006732	High	Low
XANTHOSINE	0.005297	0.006732	High	Low
QUEUINE	0.005399	0.00679	High	Low
PROTOHEME	0.005507	0.006812	Low	High
3-HYDROXY-L-KYNURENINE	0.005528	0.006812	High	Low
L-ARABITOL	0.005782	0.007054	High	Low
BIOTIN	0.005985	0.007162	High	Low
56-DIHYDROXYINDOLE-2-CARBOXYLATE	0.005988	0.007162	High	Low
FARNESYL-PP	0.006338	0.007507	High	Low
ERYTHROSE-4P	0.00646	0.00751	High	Low
CORTICOSTERONE	0.006482	0.00751	Low	High
CHOLINE	0.006571	0.00751	High	Low
5-PHOSPHORIBOSYL-5-AMINOIMIDAZOLE	0.006586	0.00751	High	Low
3-HEXAPRENYL-45-DIHYDROXYBENZOATE	0.006663	0.007526	Low	High
5Z8Z11Z14Z17Z-EICOSAPENTAENOATE	0.007098	0.00792	Low	High
ADENOSINE	0.007141	0.00792	Low	High
N-ACETYL-BETA-GLUCOSAMINYLAMINE	0.007898	0.008638	Low	High
5-10-METHENYL-THF	0.007939	0.008638	High	Low
ETHANOL-AMINE	0.008001	0.008638	High	Low
CADAVERINE	0.008281	0.008848	High	Low
DEOXYADENOSINE MONOPHOSPHATE	0.00834	0.008848	High	Low
ALLYSINE	0.008677	0.009126	High	Low
3-HEXAPRENYL-4-HYDROXY-5-METHOXYBENZOATE	0.008884	0.009264	Low	High
3-MERCAPTO-PYRUVATE	0.009023	0.009329	Low	High
DEOXYURIDINE MONOPHOSPHATE	0.009472	0.009659	High	Low
N6N6N6-TRIMETHYL-L-LYSINE	0.0095	0.009659	High	Low
HISTAMINE	0.009666	0.009746	High	Low

3-OXO-CHOLYL-COA	0.009759	0.009759	High	Low

Supplementary Table S2: Negative ionization annotated compounds with their correspondent p.value, FDR and their expression.

Compound	p.value	FDR	CAN	NOR
T2-C4-DECADIENYL-COA	5.45E-08	1.12E-05	High	Low
NICOTINAMIDE ADENINE DINUCLEOTIDE PHOSPHATE	2.23E-07	2.22E-05	High	Low
SEDOHEPTULOSE	3.23E-07	2.22E-05	High	Low
GLYCERATE/XANTHINE	5.51E-07	2.84E-05	High	Low
DTDP-DEOH-DEOXY-GLUCOSE	9.15E-07	2.92E-05	High	Low
OXIDIZED-GLUTATHIONE	9.71E-07	2.92E-05	High	Low
CMP-N-ACETYL-NEURAMINATE	1.17E-06	2.92E-05	High	Low
CARBAMYUL-L-ASPARTATE	1.37E-06	2.92E-05	Low	High
CREATINE/GUANINE	1.67E-06	2.92E-05	High	Low
LACTATE	1.82E-06	2.92E-05	High	Low
N-METHYLPHENYLETHANOLAMINE	1.83E-06	2.92E-05	High	Low
UDP-N-ACETYL-D-GLUCOSAMINE	1.84E-06	2.92E-05	High	Low
HEXOSE	3.77E-06	5.20E-05	High	Low
N-ACETYL-D-GLUCOSAMINE-P	3.79E-06	5.20E-05	High	Low
5-P-BETA-D-RIBOSYL-AMINE	4.76E-06	6.12E-05	High	Low
CDP-ETHANOLAMINE	7.47E-06	8.84E-05	High	Low
URACIL	7.72E-06	8.84E-05	High	Low
L-ERYTHRO-4-HYDROXY-GLUTAMATE	8.98E-06	9.25E-05	High	Low
ACETYL-GLUTAMATE	9.27E-06	9.25E-05	High	Low
3-P-SERINE	9.43E-06	9.25E-05	High	Low
4-PHOSPHONOOXY-THREONINE	1.16E-05	0.000107	High	Low
DTDP-D-GLUCOSE	1.19E-05	0.000107	High	Low
THIAMINE	1.27E-05	0.000109	High	Low
CYTIDINE 5′-DIPHOSPHOCHOLINE	2.20E-05	0.000174	High	Low
ADENOSINE_DIPHOSPHATE_RIBOSE	2.72E-05	0.000201	High	Low
5-PHOSPHO-RIBOSYL-GLYCINEAMIDE	2.82E-05	0.000201	High	Low
2-HYDROXY-3-KETO-5-METHYLTHIO-1-PHOSPHOP	2.98E-05	0.000205	High	Low
5′-DEOXYADENOSINE	4.33E-05	0.00028	High	Low
D-TRYPTOPHAN	4.36E-05	0.00028	High	Low
CANAVANINE	5.41E-05	0.000328	High	Low
5-METHYLTHIOADENOSINE	5.53E-05	0.000328	High	Low
CYSTEINE	5.74E-05	0.000328	Low	High
DIHYDROLIPOAMIDE	6.05E-05	0.000337	Low	High
INOSINE MONOPHOSPHATE	6.28E-05	0.00034	High	Low
METHYL-GLYOXAL	6.91E-05	0.000365	High	Low
GLUTATHIONE	9.11E-05	0.000469	High	Low
ADENOSINE DIPHOSPHATE GLUCOSE	9.64E-05	0.000482	High	Low
5-HYDROXY-TRYPTOPHAN	9.83E-05	0.000482	High	Low
PTEROATE	0.00011528	0.000537	High	Low

INOSINE	0.00011834	0.000537	High	Low
L-CITRULLINE	0.00011996	0.000537	High	Low
HISTIDINE	0.00013482	0.000567	High	Low
INDOLE_ACETALDEHYDE	0.00013498	0.000567	High	Low
S-LACTOYL-GLUTATHIONE	0.00014069	0.00057	High	Low
S-ACETYLDIHYDROLIPOAMIDE	0.0001493	0.000591	High	Low
PROSTAGLANDIN-H2	0.00015987	0.000597	Low	High
CHOLESTEROL	0.00016127	0.000597	High	Low
MEVALONATE	0.00017347	0.000627	High	Low
5Z8Z11Z14Z17Z-EICOSAPENTAENOATE	0.00021243	0.000718	Low	High
2′-DEOXYURIDINE 5′ DIPHOSPHATE	0.00021375	0.000718	High	Low
N-ACETYL-BETA-GLUCOSAMINYLAMINE	0.00021596	0.000718	High	Low
3-KETOLACTOSE	0.00023641	0.000773	High	Low
NICOTINATE_NUCLEOTIDE	0.00024477	0.000787	High	Low
5-10-METHENYL-THF	0.00024825	0.000787	High	Low
BIOTIN	0.00025384	0.000792	High	Low
MORPHINONE	0.0002999	0.000883	High	Low
NICOTINAMIDE_RIBOSE	0.00030015	0.000883	High	Low
5-PHOSPHORIBOSYL-N-FORMYLGLYCINEAMIDINE	0.00031077	0.000902	High	Low
ASCORBATE/XYLULOSE 5P	0.0003212	0.000919	High	Low
GLYCEROL-3P	0.00035641	0.000992	High	Low
PROTOHEME	0.0003659	0.001005	High	Low
DEOXYINOSINE	0.00039718	0.001063	High	Low
CYTIDINE DIPHOSPHATE	0.00040909	0.001067	High	Low
PHOSPHORIBOSYL-FORMAMIDO-CARBOXAMIDE	0.00040914	0.001067	High	Low
VITAMIN_D3	0.00043259	0.00111	High	Low
PHENYLALANINE	0.00043629	0.00111	High	Low
TAURINE	0.00049017	0.001221	High	Low
SERINE	0.00049721	0.001221	High	Low
ERYTHROSE-4P	0.00050911	0.001234	High	Low
COPPER	0.00055075	0.001289	Low	High
HEXOSE-P	0.00055995	0.001296	High	Low
OXALO-SUCCINATE	0.00074864	0.001708	Low	High
GLC-D-LACTONE	0.00075476	0.001708	High	Low
4-GUANIDO-BUTYRAMIDE	0.00076288	0.001708	High	Low
AMINO-PARATHION	0.00078608	0.001723	High	Low
SEROTONIN	0.00084076	0.001823	High	Low
UDP-GLUCURONATE	0.00087314	0.001854	High	Low
NIACINAMIDE	0.00093775	0.001971	High	Low
TETRAHYDROBIOPTERIN	0.0010006	0.002061	High	Low
GLUTAMINE	0.0010363	0.002114	High	Low
N-ACETYL-5-METHOXY-TRYPTAMINE	0.0010911	0.002204	High	Low
S-HYDROXYMETHYLGLUTATHIONE	0.0012225	0.002422	High	Low

INOSINE 5′-DIPHOSPHATE	0.0012963	0.002473	High	Low
4-HYDROXYPHENYLLACTATE	0.0014687	0.002776	Low	High
3-MERCAPTO-PYRUVATE	0.001513	0.002834	High	Low
4-TRIMETHYLAMMONIOBUTANAL	0.0015448	0.002867	High	Low
TYROSINE	0.0015893	0.002923	High	Low
L-ORNITHINE	0.0016323	0.002976	High	Low
AMMONIUM PERSULFATE	0.0016934	0.003043	High	Low
LINOLENIC_ACID	0.0016989	0.003043	Low	High
PARAOXON	0.0017711	0.003145	High	Low
RIBOSE-15-BISPHOSPHATE	0.0018845	0.003318	High	Low
BILIVERDINE	0.0021137	0.00369	High	Low
PROLINE	0.0022008	0.003778	High	Low
ADENOSINE	0.0022621	0.00382	Low	High
L-CANALINE	0.0023868	0.003939	High	Low
2-KETO-GLUTARAMATE	0.00239	0.003939	High	Low
PHOSPHORYL-CHOLINE	0.0024594	0.004021	High	Low
CHLORDECONE	0.0024811	0.004025	Low	High
DEOXYURIDINE MONOPHOSPHATE	0.002537	0.004083	High	Low
ADENOSYL-HOMO-CYS	0.0025664	0.004098	High	Low
DODECANOATE	0.0033867	0.005285	Low	High
GLYCOCHOLIC_ACID	0.0036353	0.005631	High	Low
BENZYL-ALCOHOL	0.0037216	0.005721	Low	High
ARACHIDONYL-COA	0.0037802	0.005768	High	Low
ADENOSINE TRIPHOSPHATE	0.0040148	0.006081	High	Low
6-PYRUVOYL-5678-TETRAHYDROPTERIN	0.0042876	0.0064	High	Low
D-SEDOHEPTULOSE-7-P	0.004498	0.006666	High	Low
AMINO-ACETONE	0.0049353	0.007262	Low	High
NICOTINAMIDE ADENINE DINUCLEOTIDE PHOSPHATE	0.0050828	0.007426	High	Low
RETINOATE	0.0056111	0.008027	Low	High
L-GLUTAMATE	0.0056741	0.008061	High	Low
GUANOSINE	0.0058062	0.008188	High	Low
N1-METHYLADENINE	0.0058803	0.008188	Low	High
DEOXYADENOSINE MONOPHOSPHATE	0.0058823	0.008188	High	Low
INOSITOL-1-3-4-TRIPHOSPHATE	0.0060757	0.0084	High	Low
INOSITOL-1456-TETRAKISPHOSPHATE	0.0064212	0.00876	Low	High
ADENOSINE DIPHOSPHATE	0.0066782	0.009051	High	Low
1-ETHYLADENINE	0.0067811	0.00913	High	Low
ADENOSINE5TRIPHOSPHO5ADENOSINE	0.0069028	0.009176	High	Low
SPERMIDINE	0.0069041	0.009176	High	Low
NICOTINAMIDE_NUCLEOTIDE	0.0070295	0.009224	High	Low
BORATE	0.0071041	0.009262	Low	High
CYSTINE	0.0072874	0.009442	High	Low
NICOTINAMIDE ADENINE DINUCLEOTIDE	0.0074747	0.009624	High	Low

ARABITOL/XYLITOL	0.0077714	0.009944	High	Low
3-ALPHA7-ALPHA12-ALPHA-TRIHYDROX	0.0080314	0.010213	High	Low
19-OXO-TESTOSTERONE	0.0082453	0.010246	High	Low
TRIPHOSPHATE	0.0083644	0.010318	High	Low
ALBENDAZOLE	0.0087635	0.010561	High	Low
TRIMETHYLOLPROPANE	0.0087666	0.010561	Low	High
CREATINE-P	0.0088979	0.010595	Low	High
GLYCINE	0.0090709	0.010739	High	Low
XANTHOSINE	0.0094545	0.011129	High	Low
PYRIDOXAL	0.010217	0.011958	Low	High
MORPHINE	0.010409	0.012114	High	Low
XANTHOSINE-5-PHOSPHATE	0.011008	0.01274	Low	High
L-CYSTATHIONINE	0.011374	0.01297	High	Low
N-ACETYL-NEURAMINATE-9P	0.011384	0.01297	High	Low
ALPHA-TOCOPHEROL	0.011589	0.013117	High	Low
PALMITATE	0.012051	0.013566	Low	High
12-DEHYDRORETICULINIUM	0.012873	0.014412	High	Low
LACTOSE/SUC	0.013514	0.015048	Low	High
L-SELENOCYSTEINE	0.013764	0.015165	High	Low
SUCROSE-6P	0.013914	0.015246	Low	High
N-ACETYL-D-MURAMATE	0.014247	0.015528	High	Low
GLYCOLITHOCHOLATE	0.014732	0.015788	High	Low
5-PHOSPHORIBOSYL-5-AMINOIMIDAZOLE	0.014791	0.015788	High	Low
URIDINE MONOPHOSPHATE	0.016446	0.017223	High	Low
SULFOR TRIOXIDE	0.016697	0.017372	High	Low
N-ACETYL-SEROTONIN	0.018146	0.018558	High	Low
PYRUVATE	0.018197	0.018558	High	Low
D-6-P-GLUCONO-DELTA-LACTONE	0.018436	0.018708	High	Low
5-P-RIBOSYL-N-FORMYLGLYCINEAMIDE	0.018866	0.018958	High	Low
